# *In Vivo* Near-Infrared Fluorescence Imaging of Apoptosis Using Histone H1-Targeting Peptide Probe after Anti-Cancer Treatment with Cisplatin and Cetuximab for Early Decision on Tumor Response

**DOI:** 10.1371/journal.pone.0100341

**Published:** 2014-06-20

**Authors:** Hyun-Kyung Jung, Kai Wang, Min Kyu Jung, In-San Kim, Byung-Heon Lee

**Affiliations:** Department of Biochemistry and Cell Biology and School of Medicine, Kyungpook National University, Daegu, Korea; BK21 Plus KNU Biomedical Convergence Program, Department of Biomedical Science, Graduate School, Kyungpook National University, Daegu, Korea; Department of Plastic Surgery, Henan Provincial People's Hospital, Zhengzhou, Henan, China; Department of Internal Medicine, School of Medicine, Kyungpook National University, Daegu, Korea; Henry Ford Health System, United States of America

## Abstract

Early decision on tumor response after anti-cancer treatment is still an unmet medical need. Here we investigated whether *in vivo* imaging of apoptosis using linear and cyclic (disulfide-bonded) form of ApoPep-1, a peptide that recognizes histone H1 exposed on apoptotic cells, at an early stage after treatment could predict tumor response to the treatment later. Treatment of stomach tumor cells with cistplatin or cetuximab alone induced apoptosis, while combination of cisplatin plus cetuximab more efficiently induced apoptosis, as detected by binding with linear and cyclic form of ApoPep-1. However, the differences between the single agent and combination treatment were more remarkable as detected with the cyclic form compared to the linear form. In tumor-bearing mice, apoptosis imaging was performed 1 week and 2 weeks after the initiation of treatment, while tumor volumes and weights were measured 3 weeks after the treatment. *In vivo* fluorescence imaging signals obtained by the uptake of ApoPep-1 to tumor was most remarkable in the group injected with cyclic form of ApoPep-1 at 1 week after combined treatment with cisplatin plus cetuximab. Correlation analysis revealed that imaging signals by cyclic ApoPep-1 at 1 week after treatment with cisplatin plus cetuximab in combination were most closely related with tumor volume changes (r^2^ = 0.934). These results demonstrate that *in vivo* apoptosis imaging using Apopep-1, especially cyclic ApoPep-1, is a sensitive and predictive tool for early decision on stomach tumor response after anti-cancer treatment.

## Introduction

Gastric cancer is the second leading cause of cancer death worldwide [1]. Single-agent chemotherapy for advanced gastric cancer includes capecitabine or 5-fluorouracil, while combination therapy includes cisplatin plus 5-fluorouracil or cisplatin plus capecitabine [2]. Unfortunately, gastric cancer has shown low responsibility to chemotherapy. The response rate of advanced gastric cancer ranges from 10–30% for single-agent therapy and 30–60% for combined chemotherapy [2]. In addition, molecular targeted drugs such as cetuximab (anti-epidermal growth factor receptor antibody) and trastuzumab (anti-Her2 receptor antibody) have been used in combination with chemotherapy, resulting in diverse response rates [3]–[5]. In the light of these low response rates, monitoring and early decision of stomach tumor response after treatment with anti-cancer drugs is therefore very important in the management of cancer therapy.

Traditionally, decision on tumor response has been performed by measuring the changes in tumor size using computed tomography (CT). Such a tumor size-based decision on tumor response, however, is usually possible at two months after the start of treatment. According to the guidelines of Response Evaluation Criteria in Solid Tumors (RECIST), when there is at least 30% reduction in tumor size, the treatment is considered as a partial response, while when there is a 20% or greater increase in tumor size, it is defined as a progressive disease [6]. To reduce the consuming of time and cost for an anti-tumor therapy, it is required to make the go/no-go decision on the therapy earlier than the current method based on tumor size measurement by CT.

Measuring the uptake of ^18^F-fluorodeoxyglucose (^18^F-FDG) by tumor using positron emission tomography (PET) imaging has enabled us to make an earlier decision on tumor response after anti-tumor therapy than size-based CT imaging. ^18^F-FDG uptake of tumor tissue is decreased by the reduction in the metabolism and burden of tumor cells after chemotherapy. However, it is known that the uptake of ^18^F-FDG mainly depends on histopathological types of gastric cancer. For example, Signet-ring cell carcinoma and mucinous adenocarcinoma uptake ^18^F-FDG at low levels due to low levels of GLUT-1 transporter [7], [8]. These features make decision on gastric cancer response by ^18^F-FDG uptake limited. In addition, some types of tumor, such as breast cancer, show metabolic flare, a temporary increase of ^18^F-FDG uptake after chemotherapy, which is difficult to discriminate it from tumor relapse [9].

When tumor cells are treated with chemotherapy and molecular targeted drugs, they generally die of apoptosis [10]–[12]. Apoptotic cell death appears to occur before anatomical change or reduction in tumor size [13], [14]. In this regards, imaging of apoptosis would enable us to decide whether tumor is responsive to a treatment at an earlier stage than does imaging of size reduction. Moreover, apoptosis directly represents tumor cell death, while ^18^F-FDG uptake represents tumor metabolism and thus indirectly represents tumor cell death. Apoptotic cells put signatures or biomarkers on their surface, such as phosphatidylserine and histone H1, that are little or absent on the surface of healthy cells [15]–[17]. Apoptosis imaging probes such as annexin V and dipicoyl zinc amide that bind to phosphatidylserine have been exploited for monitoring tumor cell apoptosis *in vivo* [15]–[17].

We have previously identified ApoPep-1 that recognized apoptotic and necrotic cells through binding to histone H1 on the surface of apoptotic cells and in the nucleus of necrotic cells, respectively [18]. ApoPep-1 has been shown to be accumulated at tumor after treatment with doxorubicin [18]. Also, it has been used for imaging myocardial cell death at an early stage after myocardial infarction for the assessment of long-term heart function [19]. For therapeutic purposes, ApoPep-1 has been employed as a targeting moiety to enhance drug and T cell delivery to tumor after induction of apoptosis by chemotherapy [20], [21]. In this study, we examined whether *in vivo* imaging signals of apoptosis obtained by the uptake of linear and cyclic (disulfide-bonded) form of ApoPep-1 at an early stage after treatment are correlated with changes in tumor volume later and are able to make an early decision on tumor response possible.

## Materials and Methods

### Synthesis and fluorescence labeling of peptides

Linear (CQRPPR) or cyclic (CQRPPRC, cyclization via disulfide bonding at amino and carboxy termini) form of ApoPep-1 peptides were synthesized and purified using high-performance liquid chromatography (HPLC) to >90% purity by Peptron Inc. (Daejeon, Korea.). Peptides were labeled with FPR675 near-infrared (NIR) fluorescence dye (Bioacts Inc., Incheon, Korea.)

### In vitro binding of peptides to apoptotic cells

SNU16 human stomach cancer cell line was purchased from KCLB (Seoul, Korea). To induce apoptosis, cells were treated with cisplatin (300 ng/ml), cetuximab (200 µg/ml), and cisplatin (300 ng/ml) plus cetuximab (200 µg/ml) in combination for 24 h. The concentrations of cisplatin and cetuximab were chosen according to the previous reports [22], [23]. After treatment, cells were incubated with 10 µM of fluorescein isothiocyanate (FITC)-conjugated linear or cyclic form of ApoPep-1 at 4°C for 1 h. As control, cells were stained with Alexa488-conjugated annexin V (Life technologies, Carlsbad, CA) for 15 min at RT. Percentages of fluorescent (peptide-bound or annexin V-bound) cells were measured by flow cytometry.

### Anti-tumor treatment of mice and tumor size measurement

All animal experiments were performed in compliance with institutional guidelines and according to the animal protocol approved by the guideline of the Institutional Animal Care and Use Committee (IACUC) of Kyungpook National University (permission No. KNU 2012-15).

Eight-week old female athymic (*nu/nu*) Balb/c mice were purchased from Orient laboratories (Seongnam, Korea) and were housed under specific-pathogen-free conditions with laboratory chow and water *ad libitum*. Stomach tumor xenografts were established by subcutaneously injecting 1 x 10^7^ SNU-16 cells in 100 µl saline into the right flank. Tumors were allowed to reach 50–60 mm^3^ of volume before randomization and initiation of treatment. Treatment of tumor-bearing mice with cisplatin and cetuximab was conducted according to a previously described protocol [24]. Mice were divided into four treatment groups (*n* = 6 per group) and treated for two weeks: 1) saline control; 2) cisplatin (5 mg/kg, intraperitoneal (i.p.) injection, once per week for total two injections); 3) cetuximab (1.5 mg/kg, i.p., twice per week for total four injections); 4) cisplatin (5 mg/kg, i.p., once per week for total two injections) plus cetuximab (1.5 mg/kg, i.p., twice per week for total four injections). One round of treatment includes the injection of cisplatin at day 1 per week and cetuximab at day 1 and day 4 per week. Changes in tumor size were measured over three weeks. Diameters of tumor were measured with automatic caliper. Tumor volumes were calculated using the formula: volume  =  (length x width x height)/2, where length, width, and height means the longest dimension, shorter dimension parallel to the mouse body, and diameter of tumor perpendicular to the length and width, respectively. Tumor weights were measured after isolation of tumor mass.

### *In vivo* NIR fluorescence imaging of tumor apoptosis

*In vivo* NIR fluorescence imaging was performed after the first and second round of treatment. Each treatment group (*n* = 6) was divided into two subgroups (*n* = 3) for imaging with linear and cyclic form of ApoPep-1, respectively. Linear and cyclic form of FPR675–labeled ApoPep-1 (1.45 mg/kg and 1.54 mg/kg, respectively; equivalent to 800 nmol/kg for each peptide) was injected through the tail vein into mice. At 90 min after administration, mice were anesthetized and subjected to imaging. NIR fluorescence (typically, between 650 and 1100 nm) is favored for *in vivo* optical imaging because of its low tissue absorption and deep tissue penetration properties [25]. The excitation/emission wavelength of the FPR675 dye used in this study was 675/698 nm. Images were taken using the eXplore Optix optical imaging system (ART Inc., Montreal, Canada). This time-domain tomography system has been shown to be more sensitive with higher detection depth and spatial resolution than a continuous wave planar imaging system [26]. The acquisition time for a whole-body scanning was 15 min per mouse. Fluorescence intensity at region of interest (ROI) was measured using a analysis software provided by the manufacturer (ART Inc.).

### Histologic analysis of apoptosis

After *in vivo* imaging, mice were euthanized and the tumors were removed and frozen quickly in O.C.T. embedding medium (Sakura Finetechnical, Tokyo, Japan). Tissues were cut into 6 µm sections and stained with DAPI (4′,6-diamidino-2-phenylindole) for nucleus counterstaining. Terminal deoxy-nucleotidyl transferase-mediated dUTP nick-end labeling (TUNEL) staining was conducted using Apoptag Red In Situ Apoptosis Detection kit according to the instructions provided by the manufacturer (Millipore, Billerica, MA). Tissue sections were observed under a fluorescence microscope (Carl Zeiss, Jena, Germany).

### Correlation analysis between fluorescence intensity and tumor volume

At 3 weeks after treatment (endpoint of experiments), tumor volumes were measured and tumors were isolated for the weight measurement. The correlation between NIR fluorescence intensity and tumor volume was evaluated by the linear regression analysis using the Graphpad software.

### Stability of peptides in the serum

Peptide stability in the serum was examined as previously described [27]. Blood from mice was collected and allowed to clot, and then serum was obtained by centrifugation at 4°C twice followed by filtration (0.22 µm pore). Peptide (100 µg in 50 µl of PBS) was incubated with 50 µl of filtered serum at 37°C for the indicated time period. The incubated samples were diluted 100-fold and fractionated by C18 reverse phase FPLC with linear gradient of acetonitrile (Vydac protein and peptide C18, 0.1% trifluoroacetate in water for equilibration, and 0.1% trifluoroacetate in acetonitrile for elution). To confirm the identity of the peak from the profiles of C18 reverse phase FPLC, each peak was collected, vacuum dried, and analyzed by mass spectrometry (MS) using an MALDI-TOF mass spectrometer (Life Technologies, Carlsbad, CA).

### Statistical analysis

The statistical significance of differences between experimental and control groups was analyzed using one-way analysis of variance (ANOVA).

## Results

### *In vitro* detection of apoptosis of stomach tumor cells using ApoPep-1 after treatment with cisplatin and cetuximab

**Figure 1:**
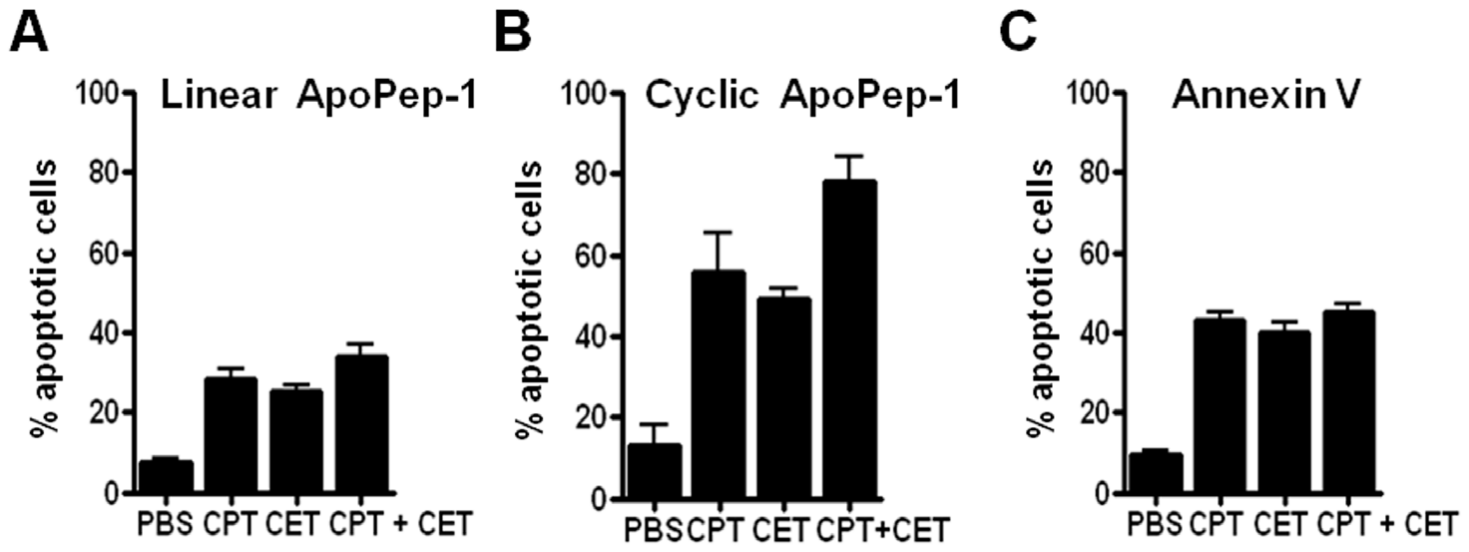
*In vitro* detection of apoptosis. Cells were incubated with cisplatin (300 ng/ml), cetuximab (200 µg/ml), and cisplatin (300 ng/ml) plus cetuximab (200 µg/ml) in combination for 24 h. Cells were harvested and incubated with FITC-labeled ApoPep-1 at 4°C for 1 h or with annexin V at room temperature for 15 min. Data represent percentages of apoptotic cells as measured by flow cytometry. (A-C) Percentages of apoptotic cells detected by linear form of ApoPep-1, cyclic form of ApoPep-1, and annexin V, respectively. PBS, phosphate-buffered saline; CPT, cisplatin; CET, cetuximab; CPT+CET, cisplatin plus cetuximab.

To examine the detection of apoptosis by ApoPep-1, stomach tumor cells were treated with cisplatin, cetuximab, and cisplatin plus cetuximab and then incubated with linear and cyclic form of ApoPep-1. Cyclic form of ApoPep-1 (CQRPPRC) was prepared by adding cysteine residue at the carboxy terminal of linear form of ApoPep-1 (CQRPPR) and cyclization through disulfide bonding. The percentages of apoptotic cells detected by the linear form of ApoPep-1 were approximately 28%, 25%, and 34% after treatment with cisplatin, cetuximab, and cisplatin plus cetuximab, respectively (Figure 1A). The percentages of apoptotic cells detected by the cyclic form of ApoPep-1 were approximately 56%, 49%, and 78% after treatment with cisplatin, cetuximab, and cisplatin plus cetuximab, respectively (Figure 1B). The percentages of apoptotic cells detected by annexin V were approximately 43%, 40%, and 45% after treatment with cisplatin, cetuximab, and cisplatin plus cetuximab, respectively (Figure 1C). These results show that the combined treatment of cisplatin and cetuximab induces apoptosis of stomach tumor cells at higher levels than the treatment of cisplatin or cetuximab alone does. Also, these results suggest that the cyclic form of ApoPep-1 more sensitively detects apoptosis of stomach tumor cells than the linear form of ApoPep-1 or annexin V does.

### *In vivo* imaging of apoptosis of stomach tumor using ApoPep-1 in response to cisplatin and cetuximab

**Figure 2:**
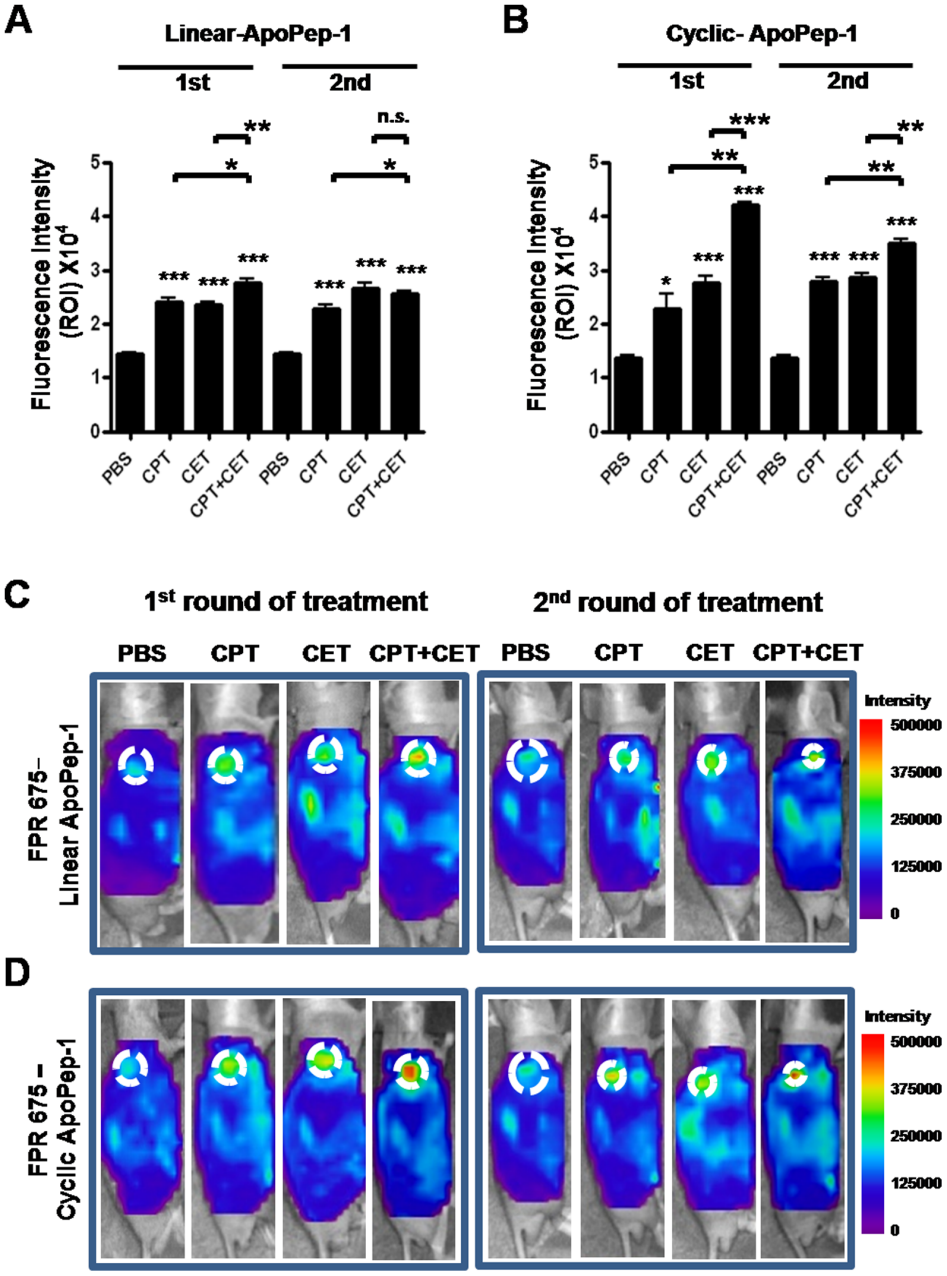
Monitoring of tumor response by *in vivo* imaging of apoptosis. SNU-16 stomach tumor-bearing mice were treated with cisplatin, cetuximab, and cisplatin plus cetuximab. After the first and second round of treatment, linear or cyclic form of FPR675 NIR fluorescence dye-labeled ApoPep-1 was intravenously injected into mice. *In vivo* NIR fluorescence images were taken at 90 min after administration. (A) (B) Quantification of NIR fluorescence signal intensity of the region of interest (ROI) in groups injected with linear and cyclic ApoPep-1. Bars represent the signal intensity at ROI obtained from three individual mice (mean ± S.D.). Asterisks represent statistical significance compared to PBS. Asterisks on brackets represent significance in difference between the two groups. * *p*<0.05, ** *p*<0.01, and *** *p*<0.001 by one-way ANOVA (*n* = 3 per group). (C) (D) Representative NIR fluorescence images by the uptake of linear and cyclic ApoPep-1 to tumor were shown. Scale bars represent normalized fluorescence intensity. Circles represent the ROI.

To examine *in vivo* detection and imaging of apoptosis of stomach tumor using ApoPep-1, we measured the fluorescence intensity at tumor by the accumulation of NIR fluorescence dye labeled-ApoPep-1 to tumor tissue after the first and second round of treatment (equivalent to one week and two weeks after the initiation of treatment, respectively). Quantification of fluorescence intensity at tumor site by either linear or cyclic form of ApoPep-1showed that the intensities were significantly higher in groups treated with cisplatin, cetuximab, and cisplatin plus cetuximab, compared to untreated control group, after the first or second round of treatment (Figure 2A, 2B). Fluorescence intensities by linear ApoPep-1 were higher in the group treated with cisplatin plus cetuximab compared to the group treated with cisplatin alone (*p*<0.05 and *p*<0.05 after the first and second round of treatment, respectively, Figure 2A) and cetuximab alone (*p*<0.01 and not significant after the first and second round of treatment, respectively, Figure 2A). Notably, fluorescence intensities at tumor site by cyclic ApoPep-1 were remarkably higher in the group treated with cisplatin plus cetuximab compared to the group treated with cisplatin alone (*p*<0.01 and *p*<0.01 after the first and second round of treatment, respectively, Figure 2B) and cetuximab alone (*p*<0.001 and *p*<0.01 after the first and second round of treatment, respectively, Figure 2B). Representative whole body fluorescence images by linear and cyclic form of ApoPep-1 were shown (Figure 2C, 2D, respectively). Little background fluorescence signals were observed in other organs, including the liver and lung (Figure 2C, 2D).

### Measurement of tumor volumes and weights after anti-tumor treatment with cisplatin and cetuximab

**Figure 3:**
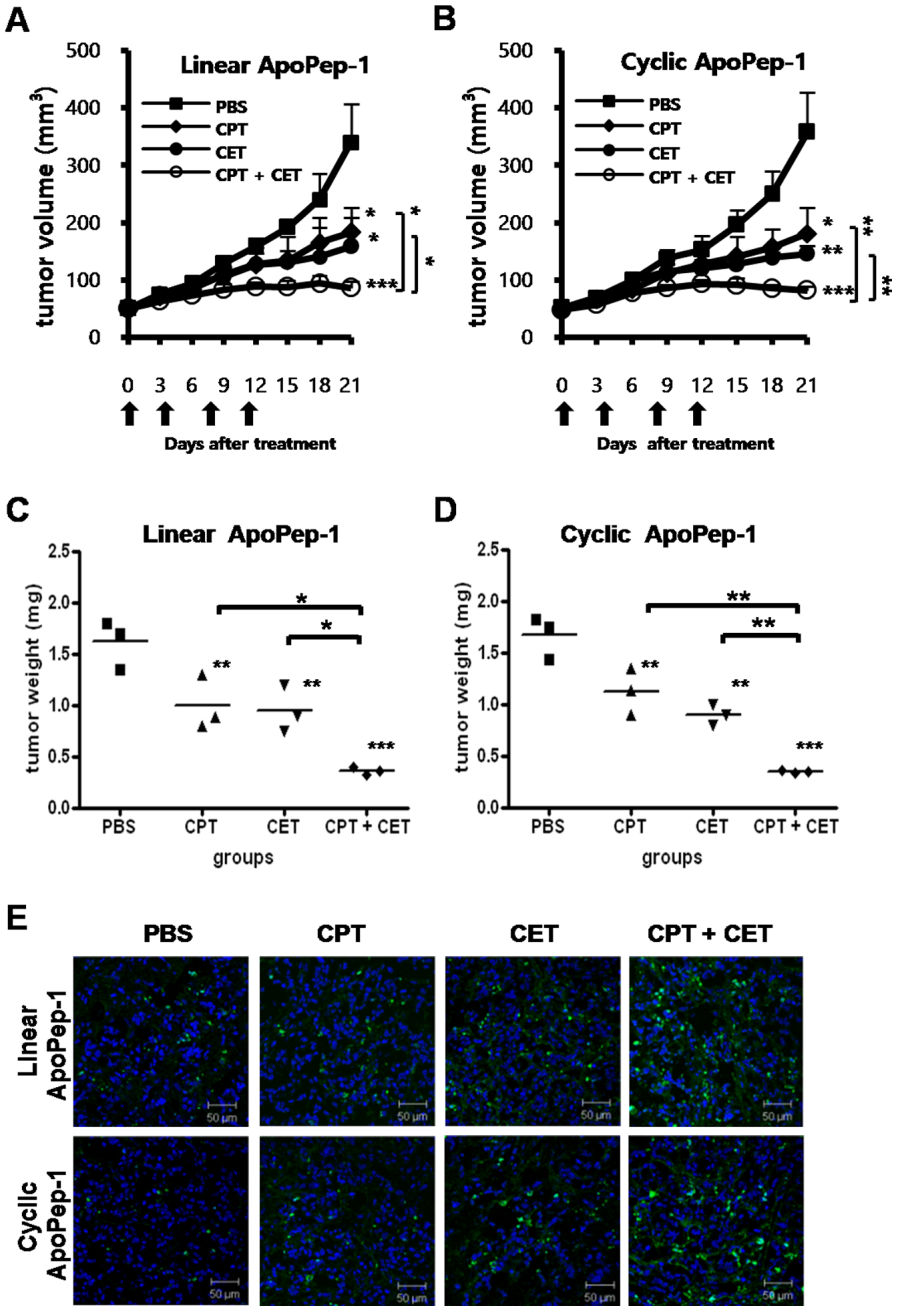
Changes of tumor volumes and weights in response to therapy. SNU-16 stomach tumor-bearing mice that were analyzed for imaging signals after the first and second round of treatment were maintained for the measurement of tumor size. (A) (B) Measurement of tumor volumes at 3 weeks after treatment. (C) (D) Measurement of weights of isolated tumor mass at 3 weeks after treatment. * p<0.05, ** *p*<0.01, and *** *p*<0.001 by one-way ANOVA. Arrows represent the time points of treatment. Asterisks represent statistical significance compared to PBS. Asterisks on brackets represent significance in difference between the two groups. (E) TUNEL staining of tumor tissues. Green, apoptotic cells; Blue, nucleus. PBS, phosphate-buffered saline; CPT, cisplatin; CET, cetuximab; CPT+CET, cisplatin plus cetuximab. Scale bars represent 50 µm.

To examine anti-tumor growth effect of cisplatin or cetuximab alone and in combination, tumor volumes and weights after treatment were measured. Treatment with cisplatin, cetuximab, and cisplatin plus cetuximab reduced tumor volumes, compared to untreated control, in the linear ApoPep-1 group (*p*<0.05, *p*<0.05, and *p*<0.001, respectively, Figure 3 A) and in the cyclic ApoPep-1 group (*p*<0.05, *p*<0.01, and *p*<0.001, respectively, Figure 3B). Combined treatment of cisplatin and cetuximab more efficiently reduced tumor volumes, compared to treatment with cisplatin or cetuximab alone, in the linear ApoPep-1 group (*p*<0.05 and *p*<0.05, respectively, Figure 3 A) and in the cyclic ApoPep-1 group (*p*<0.01 and *p*<0.01, respectively, Figure 3B).

Similar pattern of changes in tumor weights after treatment with cisplatin, cetuximab, and cisplatin plus cetuximab compared to untreated control were observed in the linear ApoPep-1 group (*p*<0.01, *p*<0.01, and *p*<0.001, respectively, Figure 3C) and in the cyclic ApoPep-1 group (*p*<0.01, *p*<0.01, and *p*<0.001, respectively, Figure 3D). Treatment with cisplatin plus cetuximab more efficiently reduced tumor weights, compared to treatment with cisplatin or cetuximab alone, in the linear ApoPep-1 group (*p*<0.05 and *p*<0.05, respectively, Figure 3C) and in the cyclic ApoPep-1 group (*p*<0.01 and *p*<0.01, respectively, Figure 3D). The levels of reduction in tumor volumes and weights after the treatment between groups injected with linear and cyclic form of ApoPep-1 were similar, and there were no differences in tumor volumes between those two groups at the time of imaging. Higher levels of apoptosis after treatment with cisplatin plus cetuximab in combination, compared to cisplatin or cetuximab alone, was further demonstrated by the TUNEL staining of the tumor tissues (Figure 3E).

### Correlation between fluorescence intensity and tumor volume

**Figure 4:**
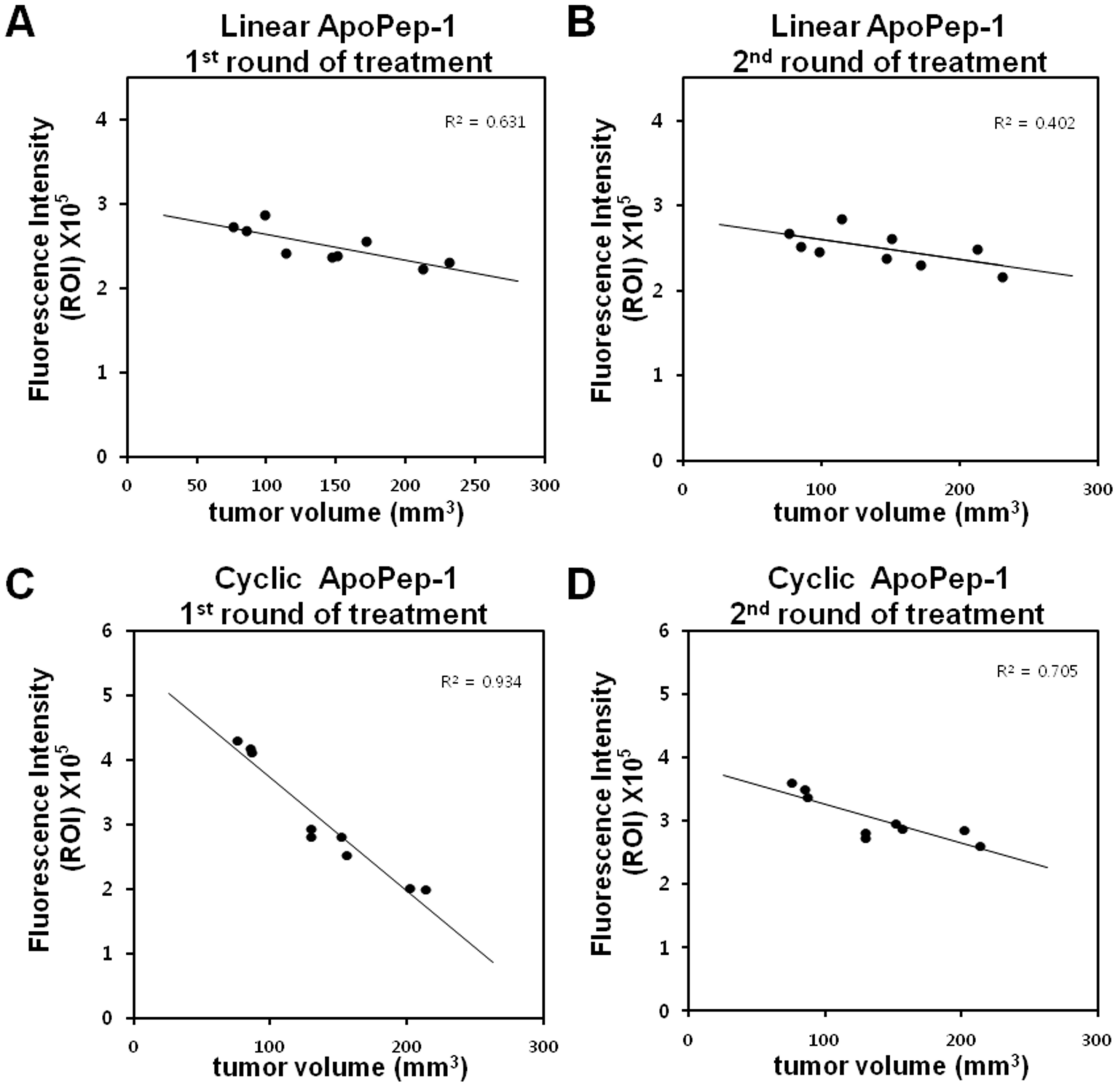
Linear regression analysis of correlation between tumor volume and fluorescence intensity. Data represent correlation between NIR fluorescence intensities obtained in Figure 2A and 2B and tumor volumes obtained in Figure 3A and 3B. (A) (B) Correlation between fluorescence intensities obtained by linear ApoPep-1 after the first and second round of treatment and tumor volumes. (C) (D) Correlation between fluorescence intensities obtained by cyclic ApoPep-1 after the first and second round of treatment and tumor volumes.

We examined the correlation between the fluorescence intensity of *in vivo* imaging of apoptosis after the first and second round of treatment (equivalent to one week and two weeks after the initiation of treatment, respectively) and tumor volume later (at 3 weeks after the initiation of treatment). The fluorescence intensities of images taken by cyclic ApoPep-1 after the first round of treatment were inversely correlated with tumor volumes with the strongest agreement (correlation coefficient r^2^ = 0.934, Figure 4C), compared to those taken by cyclic ApoPep-1 after the second round of treatment (r^2^ = 0.705, Figure 4D) and by linear ApoPep-1 after the first (r^2^ = 0.631, Figure 4A) and second round of treatment (r^2^ = 0.402, Figure 4B).

### Stability of linear and cyclic ApoPep-1 in the serum

**Figure 5:**
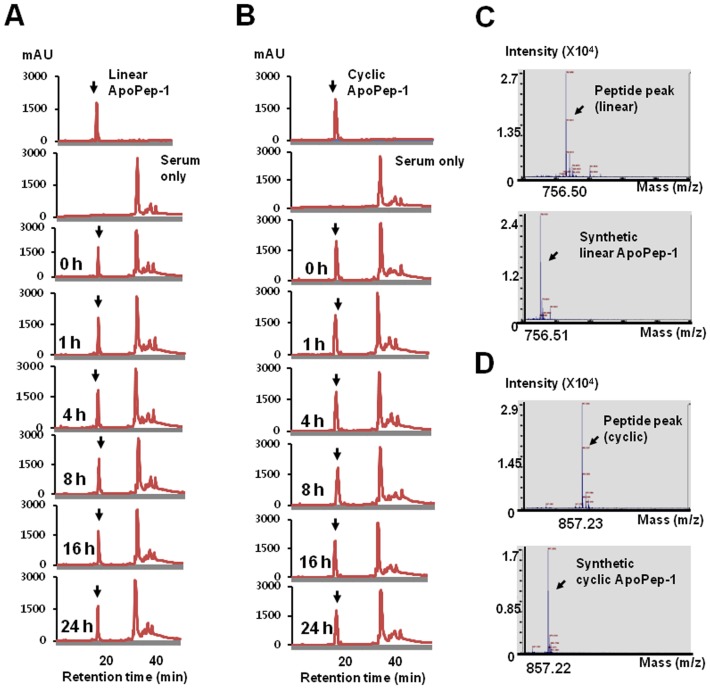
Stability of linear and cyclic ApoPep-1 in the serum. Linear and cyclic ApoPep-1 peptides were incubated with mouse serum at 37°C for the indicated time periods. (A) (B) Linear and cyclic form of ApoPpep-1 samples and mouse serum were fractionated by C18 reverse-phase FPLC. Y axis represents the absorbance unit at 215 nm. Each peptide peak was indicated by an arrow and separable from serum peaks. (C) (D) MS spectrum of the linear and cyclic peptide peak collected from 24 h FPLC fraction and synthetic linear and cyclic ApoPep-1.

We examined whether higher levels of imaging signals by the cyclic ApoPep-1 compared to those of the linear ApoPep-1 was due to the difference in serum stability of peptides. After incubation of the linear and cyclic form of ApoPep-1 with mouse serum up to 24 h, the amount of the peptide remaining in the serum was analyzed. The peptide peak was separable from nonspecific peaks of serum and the amount of linear and cyclic form of peptide remaining in the serum, as calculated by peak area, was not significantly changed up to 24 h (Figure 5A and 5B, respectively). MS analysis of each peptide peak confirmed the identity of the linear (Figure 5C) and cyclic (Figure 5D) form of ApoPep-1. These results suggest that both the linear and cyclic forms of ApoPep-1 are stable in the serum up to 24 h with no difference in stability within the incubation time period.

## Discussion

Here we showed that the *in vivo* imaging of apoptosis by the uptake of ApoPep-1 to tumor at an earlier stage (one week after treatment) could predict the stomach tumor response and subsequent reduction in tumor volume at a later stage (three weeks after treatment). In addition, the cyclic form of ApoPep-1 showed higher levels of *in vitro* binding to apoptotic cells and *in vivo* imaging signals and more remarkable difference between cisplatin or cetuximab alone and cisplatin plus cetuximab in combination than the linear form of ApoPep-1 did (more sensitive detection). The intensities of imaging signals taken by the cyclic ApoPep-1 after the first round of treatment showed closer correlation with changes in tumor volume than did those by linear ApoPep-1 (more specific detection). These results indicate that the imaging of apoptosis using the cyclic ApoPep-1 could be a useful tool for an earlier decision of stomach tumor response after anti-cancer treatment than currently available tool based on the tumor size measurement by CT scan.

Apoptosis imaging probes that recognize different biomarkers have been labeled with diverse imaging moieties and exploited for monitoring of tumor response after anti-cancer treatment. For example, fluorescence dye-labeled annexin V was given into colon tumor-bearing mice after one week of cetuximab treatment and showed a peak accumulation at 24 h after intravenous administration, which was associated with a decrease of epidermal growth factor uptake and activation of caspase-3 [28]. ^3^H-labeled butyl-2-methyl-malonic acid that binds to anionic phospholipid was given into colon tumor-bearing mice at 24 h after chemotherapy and was accumulated at tumor by 2 h after injection, which was accompanied with a decrease of tumor weights [29]. Fluorescence dye-labeled caspase activity-based peptide probe was given into mice bearing colon tumor at 12 h after treatment with Apomab to induce apoptosis, which in turn showed fluorescence signals at 50 min after injection [30]. ^124^I-labeled phosphatidylserine antibody was injected into mice bearing prostate tumor 24 h after treatment with chemotherapy or radiotherapy, in which images were taken 48 h after antibody injection and showed increased uptake of the antibody at tumor and inverse correlation between antibody uptake and the change in tumor volume (r^2^ = 0.85) [13]. Compared to the previous reports, our results suggest that ApoPep-1 is a promising probe in terms of fast uptake rate (2 h) and close correlation with tumor volume change (r^2^ = 0.934).

A cyclic form of a peptide is generally more stable against degradation by protease and more selective in target binding than its linear form [31]. A cyclic form of RGD peptide, for example, shows improved stability against pH changes [32]. Clinical trials as a potential angiogenesis inhibitor are undergoing with cyclic form of RGD peptide [31]. In some cases, however, linear form of a peptide showed better binding activity and imaging signals [31]. In the present study, we compared linear and cyclic form of ApoPep-1 to see which form shows better activity in detecting apoptosis. We found that cyclic form of ApoPep-1 was more sensitive in binding and detecting apoptotic cells than its linear form. Why did the cyclic form of ApoPep-1 show better *in vitro* binding and *in vivo* detection activity on apoptotic cells? We examined the stability of peptides in the serum. It has been previously described that ApoPep-1 was stable up to 2 h in the serum [33]. In this study, we extended the incubation time period and found that both the linear and cyclic form of ApoPep-1 were stable in the presence of serum until 24 h. This suggests that the difference in serum stability does not contribute to the enhanced targeting activity of the cyclic form of ApoPep-1 over the linear form of ApoPep-1. An alternative explanation may be that the formation of constrained structure by disulfide bonding may lead to more favorable binding to apoptotic cells by the cyclic ApoPep-1 over its linear form.

In addition to fluorescence dyes, ApoPep-1 may be labeled with radioisotopes, such as ^123^I, ^18^F, and ^68^Ga, through chemical linkers and be used as a probe for single photon emission computed tomography (SPECT) or PET imaging. As a future direction, PET imaging of apoptosis using ^18^F-labeled linear or cyclic ApoPep-1 remains to be investigated for monitoring of tumor response. ApoPep-1-based imaging of apoptosis would be useful in consideration of therapeutic strategies in clinics and contribute to the development of new anti-cancer therapeutics.
